# Arsenic in Cancer Treatment: Challenges for Application of Realgar Nanoparticles (A Minireview)

**DOI:** 10.3390/toxins2061568

**Published:** 2010-06-21

**Authors:** Peter Baláž, Ján Sedlák

**Affiliations:** 1 Institute of Geotechnics, Slovak Academy of Sciences, 043 53 Košice, Slovakia; Email: peter.balaz@saske.sk; 2 Cancer Research Institute, Slovak Academy of Sciences, 833 91 Bratislava, Slovakia

**Keywords:** arsenic sulfide, realgar, cancer, nanoparticle, milling

## Abstract

While intensive efforts have been made for the treatment of cancer, this disease is still the second leading cause of death in many countries. Metastatic breast cancer,  late-stage colon cancer, malignant melanoma, multiple myeloma, and other forms of cancer are still essentially incurable in most cases. Recent advances in genomic technologies have permitted the simultaneous evaluation of DNA sequence-based alterations together with copy number gains and losses. The requirement for a multi-targeting approach is the common theme that emerges from these studies. Therefore, the combination of new targeted biological and cytotoxic agents is currently under investigation in multimodal treatment regimens. Similarly, a combinational principle is applied in traditional Chinese medicine, as formulas consist of several types of medicinal herbs or minerals, in which one represents the principal component, and the others serve as adjuvant ones that assist the effects, or facilitate the delivery, of the principal component. In Western medicine, approximately 60 different arsenic preparations have been developed and used in pharmacological history. In traditional Chinese medicines, different forms of mineral arsenicals (orpiment—As_2_S_3_, realgar—As_4_S_4_, and arsenolite—arsenic trioxide, As_2_O_3_) are used, and realgar alone is included in 22 oral remedies that are recognized by the Chinese Pharmacopeia Committee (2005). It is known that a significant portion of some forms of mineral arsenicals is poorly absorbed into the body, and would be unavailable to cause systemic damage. This review primary focuses on the application of arsenic sulfide (realgar) for treatment of various forms of cancer *in vitro* and *in vivo*.

## 1. Introduction

While intensive efforts have been made for the treatment of cancer, this disease is still the second leading cause of death in many countries. Nearly 10.9 million new cases were diagnosed in the year 2005. In spite of enormous effort, the mortality rate due to cancer did not change in the past five to six decades [1]. Metastatic breast cancer, late-stage colon cancer, malignant melanoma, and other forms of cancer are still essentially incurable in most cases. The past quarter century of outstanding progress in fundamental cancer biology has not translated into even distantly comparable advances in the clinical practice [2]. The effectiveness of cancer therapy is limited by the rapid removal of anticancer agents from the circulation by metabolism or excretion, by the inability to access and penetrate the target cancer cells, or by nonspecific uptake by sensitive normal cells and tissues [3].

A strategy could be to associate anticancer agents with nanoparticles so as to overcome mechanisms of resistance and to increase the selectivity of such agents towards cancer cells, while reducing the drugs’ toxicity towards normal cells and issues. If designed properly, nanoparticles (defined usually as materials with a size < 100 nm) may act as drug vehicles that are able to target tumor tissues or cells, to a certain extent, while protecting the agent from premature activation during its transport [4]. Because their properties differ from those of their bulk counterparts, nanoparticles offer a range of potential applications in cancer treatment, especially in the targeting and imaging or cells or tissues.

This review primary focuses on the application of arsenic sulfide (As_4_S_4_, realgar, REA) for the treatment of various forms of cancer *in vitro* and *in vivo*.

## 2. Medicinal Use of Arsenic Compounds

Several papers describing the effect of arsenic derived compounds for medicinal purposes have been published recently [5,6,7,8].

### 2.1. History

Arsenic compounds had a long Janus-type interaction with humanity. On one hand, they have been extensively utilized, but on the other hand, their poisonous properties have caused misery and many deaths. Because of their significant properties, arsenic compounds have been used as therapeutic agents for more than 2400 years [5]. In early times of the East, a mixture of orpiment, slaked lime (CaO), and water was favored as a depilatory. Moreover, a paste of realgar was recommended by Hippocrates for treatment of ulcers. Arsenic compounds have also been used to treat the plague, malaria, and cancer. Arsenides and arsenic salts were key ingredients in antiseptics, antispasmodics, antiperiodics, caustics, cholagogues, hematinics, sedatives, and tonics. Approximately 60 different arsenic preparations have been developed and distributed during the history [7]. 

Among inorganic compounds of arsenic, Fowlers’ solution (potassium bicarbonate-based solution of arsenic oxide As_2_O_3_) was used in the treatment of asthma, chorea, eczema, pemphigus, pernicious anemia, psoriasis, Hodgkin’s disease, and leukemia. However, in the early 20th century, this form of cancer management was supplanted by radiation therapy and cytotoxic chemotherapy [8]. 

### 2.2. Arsenic oxide As_2_O_3_ (arsenolite, ATO) and tetraarsenic oxide As_4_O_6_ (TAO)

In Chinese traditional medicine, ATO has been applied for more than 500 years. Pishang, or white arsenic, which essentially contained ATO, was used originally in the treatment of certain skin diseases and asthma, and to promote the healing of surgical wounds [9]. Based on the principle in Chinese traditional medicine of “using a toxic agent against a toxic agent”, in the early 1970s, a group of clinical researchers at Harbin Medical University began to treat some types of cancer with ATO. These initial treatments were followed by a careful clinical trial at the Shanghai Second Medical University, which documented remarkable clinical efficacy in patients with newly diagnosed and relapsed acute promyelocytic leukemia (APL) [8]. Patients were treated daily with 10 mg of intravenous ATO infused over two to three hours. This monotherapy produced complete remission in six (85.7%) of seven patients presenting with de novo APL [10]. The pivotal studies in China have been followed by investigations in the United States. The induction of complete remission with low doses ATO in APL patients has been confirmed [11]. The exhaustive list of clinical trials employing arsenic-based cancer drugs in hematological malignancies and in solid tumors was published [6], while detailed overviews about the signal transduction pathways and the transcription factors triggered by ATO in leukemic cells are reviewed in [12]. It was suggested that alteration of cellular function and intracellular signal transduction pathway modulations by arsenical drugs may result in the induction of apoptosis, inhibition of growth and angiogenesis, and the promotion of differentiation [7]. Recently, the proteolytic degradation of the oncoprotein, PML-RARA, as a general strategy for the eradication of APL with ATO, due to the rapid clearing of leukemia initiating cells, was presented [13]. 

In 2003, novel orally administrable angiogenesis inhibitors were introduced by a Korean-American team, and the ability of TAO to suppress several steps of the angiogenic process both *in vitro* and *in vivo* was documented [14]. It was shown that this agent significantly decreased the proliferation, migration, invasion, and tube formation of endothelial cells induced by the angiogenic factors. In summary, TAO appears to be a novel antiangiogenic and antimetastatic chemical agent that can be orally administered [14]. 

### 2.3. Arsenic sulfide As_4_S_4_ (realgar, REA) on scene

The use of arsenic sulfide As_4_S_4_ (realgar, REA) for battling cancer, in spite of a long tradition of arsenic application for medicinal treatment, is seen only in a few countries. With the exception of China—where application of REA can be traced for a very long time—*in vitro* experiments were observed only in Korea [15], Singapore [16], and Slovakia [15,17].

### 2.4. Chinese traditional medicine

Realgar (*Xiong Huang*) is listed in the modern Chinese Pharmacopoeia and is suggested to be made into pills or powders for use, and is taken at a dosage of 150–300 mg each time, with long-term administration being avoided. The discovery that indigo (a purple dye obtained from certain plants) has a component, indirubin, which is clinically effective for leukemia, was made in the early 1960s, when researchers at the Institute of Hematology, Chinese Academy of Sciences (Beijing) noted that an herbal formula prescribed by Chinese doctors appeared to be producing good results in many APL patients. A modified version of the traditional formula, which includes both indigo and REA with Salvia miltiorrhiza, and tanshinone IIA as major active ingredients, was developed at this Institute [18]. 

The fundamental problem in REA application is its low solubility. REA in *Niuhuang Jiedu Pian*, a common preparation for the common cold, has a low solubility in water, and only 4% is bioavailable in physiological gastric juice or intestinal fluid [19]. The average total arsenic concentration in a *Niuhuang Jiedu Pian* is approximately 7 ± 1% (*i.e.*, 70,000 ppm), corresponding to 28 mg of arsenic per pill; of this only 1 mg of arsenic finds its way into the blood stream, and 40% of this absorbed arsenic (0.4 mg) is excreted in urine [19]. To overcome the low solubility and poor bioavailability, realgar particles have been prepared and applied in nanosized dimensions (see coming text). The average rate of DNA damage in the total body is represented by the urinary level of 8-OH-dG  (8-hydroxy-2′-deoxyguanosine), and is the most accepted biomarker of oxidative DNA damage. A recent study [20] reveals that arsenite caused significantly higher urinary 8-OH-dG levels than both realgar and orpiment on exposure to the same amount of compound. As the effectiveness of subsequent DNA repair determines the outcomes of treatment, significant differences may exist between species, as well as between individuals, due to inter-individual variation in DNA damage repair [21].

Both efficacy and side effects need to be considered in evaluating a drug. As described in previous paragraphs, ATO is an effective drug in patients with APL, despite the occurrence of some severe side effects. REA is almost insoluble in aqueous solution at neutral and slightly acidic conditions, and consequently will exhibit much less toxicity and better tolerance, in comparison with patients treated with ATO. NMR-based metabolomic analysis of urine, serum, and aqueous extracts of the liver tissue of Wistar rats fed with REA highlighted a number of complex disturbances in the endogenous metabolite profiles. Altered transmethylation, hypoglycemia, hyperlipoidemia, suspected reduction of intestinal microflora, and oxidative injury of the liver are related to realgar induced biochemical pathway perturbation [22]. It will be shown that REA is also effective for patients who relapsed after treatment with ATRA or chemotherapy. Since smaller amounts of realgar are needed for the induction of differentiation, than for apoptosis, the differentiation therapy deserves to be developed as a complement to other current therapies [23]. 

### 2.5. Solid state properties

Realgar is a red compound that exists in several crystalline forms. At least two polymorphs of As_4_S_4_ (sometimes referred as As_2_S_2_ or AsS) have been reported to date. The room temperature stable α-As_4_S_4_ phase is structurally identical to mineral realgar [24]. A high-temperature phase, β-As_4_S_4_ can be obtained by heating realgar above 260 °C. The β-phase can also be obtained by high-energy milling [25,26]. The β-phase is stable and slowly reconverts to the α-phase upon cooling. Upon exposure to visible light, both α- and β-phase transform from red materials into bright yellow pararealgar [27,28,29,30]. Both As_4_S_4_ phases consist of covalently bonded As_4_S_4_ cage-like molecules that have different crystal structures. The molecules are held together by weak van der Waals interactions [29]. Recently, the reaction mechanism underlying the photoinduced linkage isomerization of discrete As_4_S_4_ molecules in realgar to its pararealgar form was observed [31]. Because of the fact that arsenic occurs naturally in many minerals and in groundwater in contact with geologic formations containing a high content of arsenic, more than 20 prokaryotes utilize arsenic as a terminal electron acceptor for energy generation that goes beyond the commonly used redox mechanisms of detoxification [32]. Among these organisms, a distinct position is held by YeAs, which produces a biogenic mineral, β-realgar [33]. Recently, the production of extracellular networks of filamentous arsenic-sulfide nanotubes by Shewanella sp. strain HN-41 was discovered [34]. The formed nanotubes are initially amorphous As_2_S_3_ that evolve with increasing time toward realgar and duranusite (AS_4_S). Upon maturation, the arsenic sulfide nanotubes behaved as metals and semiconductors in terms of their electrical and photoconductive properties, respectively. 

### 2.6. Contemporary efforts

A pilot report from the Beijing University Institute of Hematology and the People’s Hospital group dealt with the treatment of APL patients with REA that appeared in the blood [35]. Moderately purified REA was mixed with an equal amount (w/w) of ground *Semen platycladi*, as an excipient, and was put into capsules containing 250 mg of As_4_S_4_. This dosage was selected on the basis of a  dose-escalation trial and animal studies. In this small study, a group of volunteer patients showed that escalation to a single dose of 60 mg/kg REA was well tolerated [36]. Hematologically complete remission (CR) was achieved in all patients with newly diagnosed APL, as well as in those with hematological relapse. Of 16 patients with newly diagnosed disease, and available cytogenetic and molecular analysis, 14 had cytogenetic and molecular CR. Treatment with well tolerated REA had only moderate side effects. Degeneration or apoptosis of APL promyelocytes was observed during REA therapy. Most urinary arsenic excretion occurred within the first 24 hours, and both blood and urine arsenic levels declined after discontinuation of REA. The results showed, for the first time, that REA treatment alone is highly effective and safe in both remission induction and maintenance therapy in patients with APL, regardless of disease stage [35]. Two years later, the first author of the described work has been granted a patent in the United States [37].

### 2.7. Mechanism of REA effect

Treatment of promyelocytic NB4 or T-leukemia CEM cells with REA induced some of the morphological features of apoptotic cells. The expression of APO 2.7, which is located at the membrane of mitochondria, was upregulated [38]. An assessment of the sensitivity of NB4 and K562 cells to REA treatment revealed a low sensitivity of K562 cells, due to high expression of Bcl-x(L), which provides resistance to REA treatment. The reversion of resistance is achieved by transfecting bcl-x(L) antisense RNA vector; cells subsequently become sensitive to realgar at clinically acceptable concentrations [39]. The expression profile analysis of NB4 cells treated with REA using 1K chip revealed the upregulation of nine genes and the downregulation of 37 genes [40]. The most pronounced effect of REA in NB4 cells—growth suppression and apoptosis—may by associated with induced reorganization of PML oncogenic domains. These domains are involved in transcriptional regulation and other different cellular functions [41]. The upregulation of proteasome PSMC2 and PSMD1 proteins can contribute to the degradation of fusion protein PML/RARa and to the increase of Hsp90. These changes are consistent with stress-related cell responses [42], or with the facilitation of interactions between abnormal protein and components of the proteasomal pathway. Examples of genes with decreased expression are as follow: MERTK, which encodes for a c-mer tyrosine kinase widely expressed in monocytes and macrophages, a regulator of G protein signaling 2 (RGS2) that belongs to a group of G0/G1 switch regulatory genes, and which is expressed in leukemia cells, with the exception of CML in the chronic phase and normal hematopoietic cells, genes that encode several ribosomal proteins, protein-tyrosine phosphatase 4A, which, as a downstream effector of p53 is involved in cell cycle regulation [43], and others.

In 2004, a study of the combined effects of REA and imatinib on CML cells and BCR-ABL oncoprotein was published [44]. Analysis of cell proliferation and clonogenic ability showed that REA and imatinib exerted synergistic effects on both K562 cells and on fresh CML cells. Both drugs under study also exhibited a synergistic effect in targeting the BCR-ABL protein. While REA triggered its degradation and imatinib inhibited its tyrosine kinase activity, their combined use led to lower enzymatic activity levels of BCR-ABL. This combination represents a new model of synergistic targeting at the molecular level and might be a promising approach for improving response rates and for resistance prevention. The expression profile of human multiple myeloma RPMI8226 cells treated with REA identifies the upregulation of 54 and the downregulation of 60 genes [45]. Upregulated genes are associated with a variety of cell functions, such as the regulation of apoptosis, cell-cell signaling, cell growth, and cell homeostasis. These genes include ones that code for the monocyte chemotactic protein MCP1, the macrophage inflammatory protein MIP-1α, several TNFα-induced proteins, and the binding protein for insulin-like growth factor IGFBP4, which is involved in the systematic and local regulation of IGF activity, along with the B-cell translocation gene BTG1, which negatively regulates cell proliferation. When comparing the extent of gene expression changes in ATO-treated RPMI8226 cells, it was found that 121 genes were upregulated and 152 down-regulated, most significantly thioredoxin-interacting protein TXNIP and activin receptor-like kinase ALK1 [46]. Results suggest that REA and ATO affect different set of genes. The additional differences in the sensitivity of cells to REA treatment may originate from differences in the tissue phenotype that the cells are derived from, because proliferation of normal human fibroblast Hs-68 cells was only slightly influenced, while high cytotoxicity in HaCaT immortalized human epidermal keratinocytes was observed [47].

CD11b expression and the NBT test confirmed that REA-induced differentiation of HL-60 cells is preceded with marked changes in ROS and catalase activity, and with small changes in superoxide dismutase activity and in levels of the reduced form of glutathione [48]. The effect of REA nanoparticles on the differentiation of HL-60 cells was synergized, enhanced, and suppressed by the inhibition of p38 MAPK, JNK, and ERK pathways, respectively [49]. The analysis of human histocytic lymphoma U937 cell death, induced by REA powder, revealed that the time- and concentration-dependent induction of apoptosis begins to decrease with an enhanced necrotic cell ratio at high REA concentrations. The use of a panel of protein kinase inhibitors demonstrates the involvement of the PI3-K/Akt signaling pathway in the downregulation of SIRT1 deacetylase activity, the subsequent activation of p53, and apoptosis induction [50]. As SIRT1 deacetylates the DNA repair factor Ku70, causing it to sequester the proapoptotic factor Bax away from mitochondria, SIRT1 inhibition can sensitize cells toward stress-induced apoptotic cell death [51]. Similar enhancement of the phosphoacetylation of the p53 paralog, p73, was observed in NB4 and K562 cells that were treated with a combination of MEK inhibitor and ATO, which promotes a p300-mediated acetylation and the p73-induced upregulation of p21 and Bax [52]. 

In paper [23], a trial has been described to compare the effect of REA and ATO on HL-60 cell differentiation, a leukemic cell line that is blocked at the level of promyelocytic differentiation [53]. The differentiation is accompanied by changes in the expression of surface antigens and in the acquisition of a number of functions indicative of cell differentiation into granulocyte-like or monocyte or macrophage-like cells [54]. ATO has been reported to induce granulocytic differentiation in HL-60 cells [55]. Although REA is similar to ATO in inducing HL-60 cell differentiation at  sub-micromolarity, the differentiation pathways are different. As shown in paper [56], the involvement of Ser/Thr protein phosphatases in the differentiation induced by REA takes a place. The total activities of PP1 (serine/threonine protein phosphatase type 1) and type 2A (PP 2A) are elevated with increasing REA concentration (in the range of 0–2.0 μM), while the differentiation extent, as indicated by the NBT reduction assay, peaked at 0.75 μM of REA. On the other hand, it was found that the inhibitors of PP1, PP2a, and PP2B (cyclosporine A and FK 506) did not have any effect on  ATO-induced differentiation in HL-60 cells [57].

These results clearly indicate that realgar As_4_S_4_ is different from arsenic oxide As_2_O_3_ in the differentiation pathway and molecular mechanism. 

### 2.8. Nanomaterials for cancer treatment: realgar case

Modification of water insoluble drugs (like REA) into ultra-fine particles with large surface areas is considered to be an efficient approach for improving bioavailability, and is practically important in the pharmaceutical industry. The application of *micronization*, if applied in a proper way, can lead to mean particle sizes of approximately 3–5 μm. However, many of the new drugs show such a low solubility that even micronization does not lead to a sufficient increase in bioavailability after oral administration [58]. Thus, by reducing particle sizes from micrometers to nanometers (*nanonization*), and thus, increasing surface area, one or more order(s) of magnitude increase in dissolution rate can be achieved [58,59,60]. Tumor vasculature is highly abnormal, proliferative, activated, and tortuous; it presents increased permeability and gaps, with pores between 350 and 800 nm in diameter, and a cutoff around 400 nm [61]. Thus preparation of nanoparticles to be 100–200 nm in diameter by any nanosizing technique gives a good predisposition for nanodrug penetration into a tumor body. While the reduction of particle sizes has been employed in the pharmaceutical industry for several decades, recent advances in milling technology have enabled the production of such nanoparticles in a reproducible manner. Nanocrystalline dispersions are prepared using the media milling processes [62,63]. The media milling process readily fractures micron-sized drug crystals into homogeneous nanoparticle dispersions.

There are a small amount of papers dealing with the application of nanorealgar (NREA) in cancer treatment. In 2001, a paper has been published which deals with the size effects of realgar particles on apoptosis in a human umbilical vein endothelial cell line ECV-304 [64]. Four different suspensions of NREA particles, with diameters in the range of 100–500 nm, which contained equivalent doses, were investigated to determine their size effects. Remarkable cytotoxic effects and induction of apoptosis confirmed by cell morphology, flow cytometry analysis, and DNA ladder presence, was achieved by exposure of cells to realgar particles of diameters of 100 and 150 nm, whereas the treatment of cells with particles of a diameter of 200 and 500 nm had only slight effects on cell viability. Authors suggested that the inhibition of angiogenesis may account for the therapeutic effect of nanosized REA particles. The concentration-dependent decrease of cancer cell line viability was observed, and the most sensitive cells for nanosized REA are HL-60 and K562 cells, followed by the more resistant multiple myeloma ARH 77 and U-266 cell lines [17].

In paper [65], the effect of long-term milling (six hours) of REA on induction of cytotoxicity in human promyelocytic leukemia HL-60 cells has been studied. Such a milling mode usually brings aggregation and agglomeration effects in solids [26]. As a post-milling procedure, authors prepared a solid dispersion using polyethylene glycol (PEG 6000) as the carrier. Based on published SEM and TEM images, one cannot be strongly convinced about the presence of nanoparticles, in spite of the author’s statement of particle size (200 nm). The described milling performance needs a more thorough, critical treatment because such a milling mode can bring contamination into realgar, as a consequence of wear from the milling media. In spite of this drawback, the paper brings new insights. It was found that treatment with NREA resulted in considerably lower cell viability compared with that of raw realgar particles. On the other hand, treatment with NREA promoted the generation of reactive oxygen species, inhibition of catalase activity, accompanied by lipid peroxidation and protein oxidation, and the loss of protein free thiols, whereas such events were not observed in HL-60 cells exposed to raw (higher diameter) REA particles. Another paper [66] shows that both NREA and raw REA particles reduced membrane fluidity in HL-60 cells, while enhancements in LDH leakage and membrane lipid peroxidation were observed in nanoparticle REA-treated cells, in comparison to control or raw REA-treated cells. In addition, nanoparticle REA significantly decreased cell viability and produced DNA ladder formation, along with the appearance of cells at the sub-G1 phase, whereas raw REA particles failed to produce characteristic apoptotic events. Treatment of HL-60 cells with REA powder filtered through a 0.22 μm filter membrane revealed that membrane lipid peroxidation was achieved at the lowest concentration of REA and LDH leakage, and DNA fragmentation occurred at higher concentrations used. Analysis of atomic force microscope images confirmed the existence of a disorganized membrane surface consisting of protruding protein clusters and confirmed the membrane toxic effect of REA in HL-60 cells [67].

The technique of cryo-milling has been applied for the first time at the National University of Singapore for the preparation of NREA samples to evaluate their *in vitro* activity and *in vivo* bioavailability [16]. The particle size of NREA ground in the presence of polyvinylpyrrolidone (PVP) or sodium dodecyl sulphate (SDS) was in the range from 176 to 243 nm, and was measured by means of a Zetasizer, while TEM images revealed a size of about 50 nm. The plausible explanation is that the particles observed by TEM were the individual realgar particles, whilst the particles apparent to the Zetasizer were the realgar particles with PVP or SDS coating(s), or both. The cytotoxic and  anti-proliferative effects of such NREA particles in human ovarian CI80-13S, OVCAR, OVCAR-3, and cervical HeLa cancer cell lines were observed. CI80-13S are most sensitive to the NREA with IC_50_ values of less than 1 μM, whereas the other cancer cell lines had IC_50_ values in a range of 2–4 μM NREA. The cytotoxic activity of the NREA particles to these human gynecological cell lines was comparable to ATO cytotoxic effects and was due to apoptosis induction. Bioavailability investigations revealed a remarkable increase in the urinary recovery of arsenic in rats after a single oral administration of the NREA particle suspension. The administered dose of arsenic recovered in urine from the NREA preparation in presence of PVP or SDS, or both, ranged from 59–79%, whereas the original REA powder gave a urinary recovery of only 25%. The improvement in bioavailability can be attributed to the realgar particles’ size reduction.

It is well known that cysteine-dependent aspartame specific proteases (caspases) are an important mediator of signal transduction in apoptosis. Both induction of apoptosis and prevention of angiogenesis enhance the anticancer effect of the transferal delivery of REA nanoparticles against mouse melanoma B16 cells. In this treatment, there seems to be no apparent *in vivo* toxic manifestation, as evaluated in terms of body weight changes, feeding behavior, skin irritation, and liver injury [68]. REA nanoparticle-induced apoptosis in U937 cells was partially prevented by inhibitors of caspase-3, -8, and -9 [69]. While inhibitors of ERK and p38 kinases failed to block cell death, the inhibitor of JNK significantly inhibits REA-induced apoptosis. The REA-induced increase of the Bax/Bcl-2 ratio and the phosphorylation of JNK are involved in U937 apoptosis. Similarly, the proliferation and viability of CML cell line K562 is affected by micromolar concentrations of REA. The REA treatment decreases Bcr-Abl protein content, although the mRNA level of the bcr-abl fusion gene does not change. Consequently the PTK activity of c-Abl and Bcr-Abl was decreased in a time- and concentration-dependent manner. Compared with CML Ph+ leukemia cells, Ph− mononuclear cells were less sensitive to REA treatment at all concentrations tested. When targeting the Bcr-Abl, the crucial factor of CML pathogenesis and the higher sensitivity of Ph+ cells to apoptosis induction suggests the therapeutic potential of REA for CML patients [70]. 

## 3. Conclusion

In traditional Chinese medicine, there is well established multicomponent characters for remedies. The combination of arsenicals with natural compounds can modify unpleasant side effects, such as cardiotoxicity or genotoxicity in normal cells [71,72] or enhance the cytotoxic effect [18]. On the other hand, the effective surface modification of insoluble arsenic particles with ligand targeted to tumor cells represents a promising tool for the enhanced hit-and-kill antitumor effect of future nanomedicine approaches to cancer patient treatment.
